# Report on the Protective Powers of Vaccination

**Published:** 1847-01

**Authors:** 


*Report on the Protective Powers of Vaccination.*—In April, 1842, the undersigned were appointed by the College, a committee to investigate the protective powers of vaccination; the phenomena resulting when those who have been already vaccinated are again subjected to the disease, and the subject of re-vaccination generally. Various obstacles, beyond their control, prevented their performing, at an earlier period, the task submitted to them, in a manner that would have proved at all commensurate with its importance; they have, however, been enabled, at length, to bring their labours to a close, the result of which is here submitted.

The first object embraced in the resolution, under which the committee was appointed, is one of acknowledged interest; it refers to the protective powers of vaccination.

The most obvious and conclusive mode of determining the degree of protection imparted by vaccination against the influence of small-pox, is to inoculate, with variolous matter, the persons who have been previously vaccinated.

At the close of 1801, the first successful efforts were made at vaccination in this city. In the early periods of this practice, until the year 1812, every mean was employed which, at the time, was deemed best calculated to determine whether the process of vaccination would afford full protection against the small-pox. The first step generally taken, after having observed the genuine character of this vaccine pock, was to insert a portion of variolous matter, and to note its progress. Where due attention had been paid to the selection and insertion of this matter, a small red pimple appeared about the third day. On the fifth day this was converted into a purulent crust, surrounded by inflammation, generally of no great extent, which, after this period, began to fade, and was rarely perceptible beyond the eighth day; it left no trace beyond the tenth. The full and distinctive character of the variolous pock was not observed on these occasions. The persons thus treated were not affected with fever, or any general disarrangement of the system, nor was any eruption observed on the skin. The persons having been submitted to this test, were next exposed to the small-pox in the most direct manner, often by placing them in the beds with those labouring under the disease, even in its most virulent form. A like immunity attended this experiment.

From repeated observations thus conducted, observing that no instance of small-pox had been communicated to those on whom the operation of vaccination had been duly performed, the profession ventured, in 1803, to announce the process as one affording equal protection with variolous inoculation.

The reasons for preferring the process of vaccination to that of variolous inoculation could not fail to be appreciated, and were strongly urged in the circulars issued for the public information.

Under the most favourable circumstances, and where every attention had been paid to insure success, many of those who had been inoculated for the small-pox were known to have fallen victims to the disease. The estimate most generally received, and founded on a large experience was, that one in four hundred of the inoculated fell victims to the process; some practitioners were stated to have been more fortunate, and to have lost not more than one in a thousand. On the other hand, of the thousands that had been vaccinated, not a death had been recorded, which could be imputed to this process. The age of the individual, or the physical condition of the other members of the family, constituted no objection to the operation, as the cow-pock could be communicated by the direct application of the virus only; while in the instance of the small-pox, the effluvia emanating from the infected, were known to impart the disease to the unprotected, and under circumstances when it was likely to prove fatal. In addition to the loss of life, caused by the small-pox, even when the result of inoculation, the mutilations which were so often consequent, even when life was preserved, must have been familiar to every one acquainted with the disease, whether casually received or by inoculation.

Unfortunately, the reputation of the Jennerian process has been materially affected by the ignorance of the persons by whom the operation has been frequently undertaken, and by the want of attention on the part of some of the profession in visiting at the proper periods, and recording the phenomena observed. The period when the areola may be expected ought never to be neglected. The evidence that may be obtained from such circumstantial records, will tend to dispel many of the idle reports so freely circulated to the disparagement of vaccination. By consulting such recorded documents, it has been clearly ascertained that the majority of the cases of small-pox ascribed to the failure of the protective process were, in reality, owing to the imperfect manner in which the operation had been conducted. There is abundant evidence recorded in medical books, of persons having suffered from small-pox more than once; where such a peculiarity of constitution exists, it cannot be a matter of surprise that the disease should be occasionally observed after vaccination, even in cases where the genuine and perfect character of the vaccine process has been fully developed. A careful examination of these histories does not exhibit any appreciable difference in the proportional number of such cases, whether after small-pox or after vaccination.

By an act of the Legislature, passed in 1811, to communicate the infection of small-pox by inoculation, or otherwise, has been prohibited, under certain pecuniary penalties. For the four succeeding years, the disease is not recorded in the bills of mortality; and the mode of most conclusively testing the protective powers of vaccination necessarily ceased. And hence the profession have, since then, been deprived of a measure so intimately connected with the inquiries to which the attention of your committee has been directed.

Touching this very important question, it must be acknowledged that the documents at command are but few; nevertheless those that have been preserved, afford the evidence that, after the lapse of three years, the vaccinated were found no longer susceptible of small-pox by inoculation. Among the persons thus treated, it has been ascertained that an infant, vaccinated within a month, resisted the variolous infection after an interval of the same number of years.

For the want of that full and comprehensive information which our more immediate resources fail to afford, it may prove acceptable if we here present the experiments made by that distinguished and accurate observer, Biot:

"In August, 1826, a number of boys, between the ages of twelve and sixteen years, were inoculated in four places with small-pox virus by Dr. Biot, one of the physicians to the hospital St. Louis, at Paris. A part of these had been vaccinated, and a part had never been protected from the small-pox. In those who had been vaccinated, the insertion of the variolous virus had no other effect than to produce a slight inflammation for two or three days at the points where the matter was introduced. Those who had never been protected were affected differently, and contracted the variolous disease."

In this stage of the inquiry, it appears not irrelevant to notice the diminished mortality from small-pox, as observed in this city, since the introduction of vaccination. Whenever that disease has appeared, it has been viewed as among the severest scourges inflicted on mankind, and the most appalling apprehensions have been entertained by every community which it has visited. The sufferings of those affected with this malady have at all times awakened the most painful and anxious feelings in friends and connections, and called forth the assiduous and unremitting care of the attendants and those administering to the sick. In seasons of epidemic small-pox, the mortality has been observed to be great. In 1721—22, in the town of Boston, five thousand seven hundred and fifty-nine persons were affected with the disease, of whom eight hundred and forty-four came to an untimely death, as publicly announced by the municipal authority of that city. This was a fearful mortality, particularly when we advert to the number of persons inhabiting Boston at that period. In the succeeding year, 1722, the census was taken, indicating a population of ten thousand five hundred and sixty-seven; if, to this, there be added the number who died of small-pox, eight hundred and forty-four, the sum will be eleven thousand four hundred and eleven, as the total population previous to the ravages of the small-pox having commenced. 'One-half of the inhabitants of the city were affected with the disease. Of those, between one-sixth and one-seventh came to an untimely death, and the city lost nearly one-thirteenth of its population.—*Zabdial Boylston, on Small-pox, p.* 33.

Of those who survived the loathsome disease, many, from the hideous aspect and mutilations caused by the disease, continued through life the objects of commiseration, and too often of disgust.

Laws have been enacted, and rigid quarantines enforced, with the view of preventing the introduction and spreading of the small-pox. In small communities, having little intercourse with other portions of the world, these efforts have proved successful for a season ; but the temporary immunity thus enjoyed has only rendered the subsequent ravages of the disease the more disastrous. The nations engaged in commerce soon experienced the futility of every attempt to insure exemption from the disease ; for though the infected were secluded, and denied all intercourse with those deemed liable to the disease, the poison was found to be frequently conveyed by means of the clothing, and articles taken from the chamber of the sick. For successive ages, the pestilence was submitted to as an inevitable evil, and allowed to extend its baneful influence, annually destroying large portions of the inhabitants of the earth, and in some instances depopulating whole districts.

The deaths from casual small-pox have varied, from circumstances not in our power to appreciate. The character of the seasons, the peculiarity of position and the nature of the intercourse between the infected and those liable to take the disease, have had their influence in determining the malignity and mortality observable in different years.

The materials having relation to the medical statistics of Philadelphia, that can be gleaned from its early history, are extremely scanty and defective. It is only within the present century. and for the period of barely forty years, that any of the reports of the deaths have been publicly made. Prior to this period the records of the interments were confined to particular religious societies, necessarily difficult of access, and, generally, ill adapted to the present purpose. Though restricted to a narrow sphere, and not embracing the entire population comprehended within the city and districts, the registers kept at the Dispensary afford a document which may constitute a proper basis to found the estimate of the advantages gained by vaccination. From the foundation of the institution to the close of 1801, including a period of sixteen years, when variolous inoculation was considered as the only protective process available against casual small-pox, fifty-one persons are stated to have died of this disease, while the entire number of deaths, from various diseases, were seven hundred and one, establishing the proportion to be seventy-three in one thousand, which accords with the most favourable estimates made in Europe, and will hardly be excepted against by those who are the least favourable to vaccination.

In 1807, by an act of the Legislatute of the State, every death within the city and districts, must be reported to the Board of Health, the certificate expressing the disease of which the person has died. From this source, clear and positive evidence may be obtained. From the annual reports during the period of four years, variolous inoculation still being permitted, it appears that the deaths from various diseases amounted to ten thousand seven hundred and forty-four, of which number, four hundred and twenty-nine were from small-pox. This affords the evidence of a considerable reduction in the proportional mortality from this disease, it being as forty in the thousand; during a period when vaccination had been practised and institutions established for its dissemination.

Since 1811, variolous inoculation has been prohibited by law. During the four succeeding years, not a death from small-pox was recorded. In 1816, there was a great influx of foreigners, particularly from the British dominions in Europe; by these persons the disease was introduced into this city, and proved fatal in that year to no less than ninety-seven persons. It was accompanied with a papular eruption, resembling, in many particulars, mild variola—affecting some of those who were believed to have been protected from small-pox by having previously undergone what was supposed to be successful vaccination; of these, no death is recorded that has come to our knowledge. In 1820, 21, 22, the city again enjoyed an exemption from this malady. This assertion is founded on the fact of no deaths from small-pox, in these years, having been reported to the Board of Health. In 1823, 24, the pestilence again afflicted our city, and the mortality from this source amounted to four hundred and eighty-four.* The fact that a large number of those who had been previously vaccinated with the greatest care, as well as many of those who had previously passed through small-pox by inoculation or otherwise, were attacked during this epidemic with a modified form of small-pox, created considerable alarm in the public mind, and directed anew the attention of the profession to an investigation of the amount of protection afforded by the vaccine disease. Upon the recurrence of small-pox in 1827, the Medical Society of Philadelphia appointed a committee to collect and report to the society all the facts within their reach, in relation to this important subject. The committee immediately addressed a circular to the physicians of the city and county, containing a series of interrogatories calculated to elicit information in relation to the protective powers of vaccination. The report of this committee, embracing the facts collected by them, was made to the society in 1828, and was of such a character as to renew the confidence of the profession in the protective powers of the vaccine infection.† Notwithstanding, it was admitted that in many instances, those who had been previously vaccinated would be liable to become affected, during the prevalence of variola, with a modified, and, usually, mild form of small-pox, yet it was shown that the public are highly benefitted by the practice of vaccination, which

*For an account of the epidemic of 1823, 24, we refer to a very able paper by Drs. Mitchell and Bell, in the North American Medical and Surgical Journ. vol. ii. p. 27 et seq.

†For a copy of this report, see North American Medical and Surgical Journal, vol. v. page 400.

was, in every sense, to be preferred to inoculation, and hence, that humanity and sound policy imperiously demanded its continuance.

In succeeding years, with occasional mitigations, Philadelphia has suffered more or less from the disease. Taking the whole period since variolous inoculation was prohibited, the mortality from all sources has amounted to one hundred and forty-three thousand and seventeen, of this number, two thousand four hundred and ninety-seven are referred to small-pox; by which it will be perceived that the proportional mortality has been reduced to eighteen in the thousand.

We have thus endeavoured to exhibit the degree of protection against small-pox afforded by the process of vaccination. The evidence adduced does not depend on individual experience, but rests on the broad basis of the public records, into which no undue bias can have entered.

It must be conceded, that the anticipations of the warm friends for vaccination, that this process would lead to the extirpation of the small-pox, have not been accomplished. In making this concession, the question necessarily arises, whether the requisite precaution, for preventing the intercourse of those affected with small-pox, and those still susceptible of the disease have been duly enforced. The remarks of one who took a lively interest in arresting the progress of this loathsome malady, appear to be particulary pertinent, and to deserve the earnest consideration of every individual desirous of removing from the community this dreadful scourge. The words of Dr. Haygarth are, "the discovery of vaccine inoculation by Dr. Jenner is the most fortunate and beneficent improvement that medical science ever accomplished. It does not, however, preclude the necessity of investigating the nature of the variolous poison, and of considering by what regulations its propagation may be prevented. In order to secure the unthinking multitude from this destructive pestilence, measures to prevent the casual *small-pox* should every where accompany vaccine inoculation; without such protecting care by the wise and hnmane part of society, this mortal malady would, for ages, lurk unheard of and unsuspected, to the annual destruction of many thousands. No town, no village, not even a single solitary house would enjoy perfect safety from danger."

The foregoing, your committee beg leave to present, as a solution of the first question submitted to them by the College.

The occurrence, year after year, of the small-pox in the city of Philadelphia, and in other communities, and the frequency with which those who had been vaccinated were attacked with a more or less severe, though generally modified form of the disease, induced many to suppose that the protective power of the vaccine infection was only temporary. The question as to the necessity of re-vaccination now arose, but whilst few practised it, many condemned it as useless, and calculated to weaken public confidence in the protective powers of vaccination. Others, however, maintained that, as there was no certain criterion by which the protective powers of the primary infection could be established with absolute certainty, re-vaccination was necessary in all cases in order to test the efficacy of the first operation. This opinion very naturally obtained importance from the fact, that few cases of modified small-pox were known to have occurred after a second vaccination, provided the insertion of the vaccine virus produced an action sufficient to prove that it had been taken up by the absorbents; also, from the disease being observed, in numerous instances, to be arrested in families, and in neighbourhoods, where those already reputed to be vaccinated were again subjected to the operation.

When vaccination has been repeated in those who have been previously subjected to the disease, it is found to produce a local affection, marked by very different degrees of intensity. In some, slight inflammation occurs on the second or third day, and then fades away. In others, various degrees of inflammation manifest themselves, followed by a pock or pustule, terminating in a brown or yellow crust, whilst, very rarely, a distinct, regular areola is produced, or the other phenomena of the genuine disease, as will fully appear from the table here annexed.

The subject of repeated vaccination has engaged a large feld of inquiry in different parts of Europe; a very full abstract of the results of which, will be found in the work of Dr. Condie on the Diseases of Children, page 458.

The second question, therefore, submitted to your committee, was to determine the phenomena resulting when those who have been already vaccinated are again subjected to the disease, and the necessity and policy of re-vaccination. It is to the first portion of this question that the committee have been obliged, necessarily, to confine their attention. It must be very evident that to test fully the necessity or propriety of re-vaccination, would either require that after large communities shall have been a second time subjected to vaccination, time be allowed in order that they may be repeatedly subjected to the influence of variolous contagion, during the epidemical visitations of small-pox, or that the direct test be resorted to of inoculating with small-pox those who have been successfully vaccinated, at different periods subsequent to the primary operation, as well as after a secondary operation, and carefully noting the comparative immunity from the impression of the variolous poison exhibited by each class of patients.

In order to obtain as wide a field of observation as was in the power of your committee they applied to the comptrollers of the public schools, soon after their appointment, for the privilege of vaccinating the scholars whose parents would consent to the operation being performed; whether they had been previously vaccinated or not; in order to accertain the degree of impression that would be made in each case by a repetition of the operation, and with a view of watching hereafter the effect or influence it may have in preventing the occurrence of small-pox in the subject of our experiments, should an epidemic of that disease again appear amongst us. But the comptrollers did not at that time consent to our request, and the investigation of the subject had to be postponed. In the early part of the present year, small-pox again made its appearance and excited considerable uneasiness in the minds of the public. Your committee entertaining the opinion that the period was propitious for a renewal of their application to the comptrollers, accordingly did so, and their consent was promptly given. Circulars were then sent to the parents of the children, attending several of the schools, requesting their permission, which was in most cases readily granted, and every facility was offered to carry into effect the object your committee had in view.

The operation was performed in nine hundred and thirty-one cases; six hundred and thirty-five of whom took on various grades of action; many of these had been vaccinated three, four, and five times before, and stood in relation to small-pox, chicken-pox, and varioloid as stated in the following account:—

Nine hundred and thirty-five persons reputed to have been protected by previous vaccination, having distinct cicatrices on the arm, or by having passed through the small-pox, were subjected to the action of the vaccine virus.

50 did not appear on the days appointed for examination.
294 exhibited no signs of inflammation from the process.
52 of these were reputed to have been previously vaccinated twice.
6 " " three times.
1 " " four times.
14 " to have had the small-pox.
36 " " varioloid.
72 " " chicken-pox.
76 had various degrees of inflammation on the fourth day, which had faded before the eighth day
15 of these were reported to have been previously vaccinated twice.
2 " " to have had the small-pox.
5 " " " varioloid.
26 " " " chicken-pox.
181 had inflammation more or less severe, accompanied by a yellow or purulent crust, on the eighth day.
30 of these were reported to have been previously vaccinated twice.
11 " " " three times.
2 " " " four times.
1 " " " seven times.
9 " to have had the small-pox.
7 " " " varioloid.
50 " " " chicken-pox.
95 had very diffusive inflammation, the pustule still moist on the 8th day.
10 of these were reported to have been previously vaccinated twice.
1 " " " three times.
1 " " " four times.
2 " to have had the small-pox.
4 " " varioloid.

23 of these were reported to have had the chicken-pox.
179 exhibited more or less inflammation and a brown crust on the 8th day,
10 of these were reported to have been previously vaccinated twice.
4 " " " three times.
1 " " " seven times.
8 " to have had the small-pox.
7 " " varioloid.
49 " " chicken-pox.
57 had a pock with various degrees of inflammation; diffused and irregular in its margin on the 8th day.
2 of these were reported to have had the small-pox.
15 " " chicken-pox.
3, viz. a boy and two girls, exhibited a well defined pock and the areola characteristic of the perfect vaccine impression as observed on the 8th day. They are stated not to have been affected with the small-pox, varioloid, or chicken-pox.

From the foregoing observations, as well as from those derived from previous practice, your committee are justified in the conclusion, 1st, that vaccination is the best preservative of human life now known against the contagion of small-pox; and although it has not answered the full expectations of its more sanguine advocates by protecting the system in all instances against at least a modified form of variola, which is the case with the small-pox itself, nevertheless, life is very generally protected by it, and humanity and sound practice imperiously call for its continuance.

2ndly. Patients who have been once fully subjected to the influence of the vaccine infection, lose, in a great degree, their susceptibility to a second infection; the susceptibility diminishing still more at every subsequent vaccination.

3rdly. That portion of the community who have been once successfully vaccinated are in the great majority of cases fully protected from small-pox or varioloid.

4thly. A second vaccination does not insure the system in every instance against an attack of varioloid; neither does second vaccination prevent an impression being made on the system by a subsequent operation.

5thly. Upon the recurrence of small-pox in a family or neighborhood, it is all important that all individuals in regard to whom there is any doubt or uncertainty as to the fact of their having been successfully vaccinated, should be subjected immediately to the operation, this being the most certain means of preventing the spread of the variolous contagion.

Your committee have not heard of a single instance of varioloid having occurred in any of the children of the public schools vaccinaed by them, notwithstanding the epidemic was prevailing at the time and subsequently.

D. Francis Condie,

Thomas T. Hewson, } *Committee.*

J. Wilson Moore.

Accompanying this report there was presented a table containing the name and age of each individual re-vaccinated by the committee, the number of times each had been previously vaccinated; noting those who had been affected with small-pox, varioloid, or chicken-pox; and the appearance of the arm on the fourth and eighth and twelfth days, with remarks on the result of each case.—*Summary of the Transactions of the College of Physicians of Philadelphia.*

